# Preliminary Consequences of Blood Pressure Management and Blood Homocysteine Levels with Perindopril in Newly Diagnosed Hypertensive Patients in the Vietnamese Population

**DOI:** 10.1155/2023/1933783

**Published:** 2023-10-18

**Authors:** Son Kim Tran, An Bao Truong, Phi Hoang Nguyen, Toan Hoang Ngo, Tuyen Long Vu, Khoa Dang Dang Tran, Phuong Minh Vo, Bao The Nguyen, Tuong Le Trong Huynh, Kien Trung Nguyen, Hung Do Tran

**Affiliations:** ^1^Faculty of Medicine, Can Tho University of Medicine and Pharmacy, Can Tho 900000, Vietnam; ^2^Department of Cardiology, An Giang Cardiovascular Hospital, Long Xuyen 880000, Vietnam; ^3^Department of Foreign Language, Can Tho University, Can Tho 90000, Vietnam; ^4^Department of Cardiology, Can Tho Central General Hospital, Can Tho 90000, Vietnam; ^5^Faculty of Nursing and Medical Technology, Can Tho University of Medicine and Pharmacy, Can Tho 900000, Vietnam

## Abstract

**Background:**

Perindopril is an ACE inhibitor that aids in both blood pressure regulation and homocysteine reduction.

**Objectives:**

Our study aimed to evaluate the results of controlling blood pressure and blood homocysteine levels by perindopril in patients with primary hypertension.

**Materials and Methods:**

A cross-sectional descriptive study with a longitudinal follow-up was conducted on 105 primary hypertensive patients treated with perindopril.

**Results:**

The results of our study showed that after 6 weeks of treatment with perindopril, the proportion of patients with the target blood pressure (BP) level accounted for 70.5%, the rate of grade 1 hypertension decreased from 61.0% to 25.7%, grade 2 blood pressure decreased from 17.1% to 3.8%, and there was no case of grade 3 hypertension. At the same time, we also found that the rate of BP control in the group of patients who controlled Hcy below a threshold of 15 *μ*mol/L was significantly higher than in the other group (*p*  <  0.05). Concerning the efficacy of decreasing homocysteine in blood, we discovered that after 6 weeks of treatment with perindopril, the proportion of patients with elevated homocysteine reduced considerably from 74.3% to 40% (*p*  <  0.05). In addition, the homocysteine concentration was 4.33 mol/L lower after treatment than before treatment (95% CI: 3.69–4.97) (*p*  <  0.05).

**Conclusion:**

Perindopril helps control blood pressure and reduces blood homocysteine levels in patients with primary hypertension.

## 1. Introduction

Hypertension is the cause of 62% of cerebrovascular disease and 49% of ischemic heart disease, according to the World Health Organization [1]. In 2018, the European Society of Cardiology (ESC) projected that 1.13 billion individuals globally suffered from hypertension. Worldwide, the prevalence of hypertension in adults ranges between 30 and 45%, with prevalence rates of 24% in men and 20% in women. It is anticipated that by 2025, the global prevalence of hypertension will increase by 15–20%, reaching roughly 1.5 billion individuals [2].

In addition to classic cardiovascular risk factors, there are additional cardiovascular risk factors such as homocysteine, C-reactive protein, fibrinogen, and lipoprotein (a) that may coexist in patients with hypertension [3–5]. Homocysteine levels in blood are regarded as an independent risk factor for cardiovascular and noncardiovascular death. Every 5 mol/l rise in blood homocysteine levels was associated with a 49% increase in total mortality and a 50% increase in cardiovascular disease mortality [6].

Numerous studies have demonstrated that lowering homocysteine levels in primary prevention improves both blood pressure and cardiovascular events [7, 8]. There have also been global investigations on the efficacy of some antihypertensive medications, particularly ACE inhibitors, on blood homocysteine levels [9, 10]. However, there have been no studies in Vietnam assessing the effect of antihypertensive medications on homocysteine levels. Consequently, we conducted this study with the following goals: to assess the efficacy of perindopril in lowering blood pressure and homocysteine levels in patients with primary hypertension.

## 2. Materials and Methods

### 2.1. Study Population

#### 2.1.1. Materials

All patients with primary hypertension visited the examination department of the Can Tho University of Medicine and Pharmacy Hospital during the period from 5/2017 to 5/2018.

#### 2.1.2. Inclusion Criteria

All patients were newly diagnosed with primary hypertension according to JNC 6 criteria [11]. This criterion is similar to the criteria for the diagnosis and grading of hypertension of ESC 2021 [12].

#### 2.1.3. Exclusion Criteria

All patients with primary hypertension had comorbidities that affect homocysteine levels such as history of liver disease, kidney disease, cerebrovascular accident, and chronic comorbidities (gout, rheumatoid arthritis, and Parkinson's disease). Subjects are currently being treated with vitamin B6, B12, and folate drugs. Diabetic patients are currently taking sulfonylurea [13]. Patients had contraindications to ACE inhibitors, i.e., pregnant or lactating women, aortic stenosis, renal artery stenosis, glomerular filtration rate <25 ml/min, and serum potassium >5.5 mmol/L [14].

### 2.2. Methods

#### 2.2.1. Study Design

A cross-sectional descriptive study with a longitudinal follow-up was conducted.

#### 2.2.2. Sample Size

**Figure 1:**
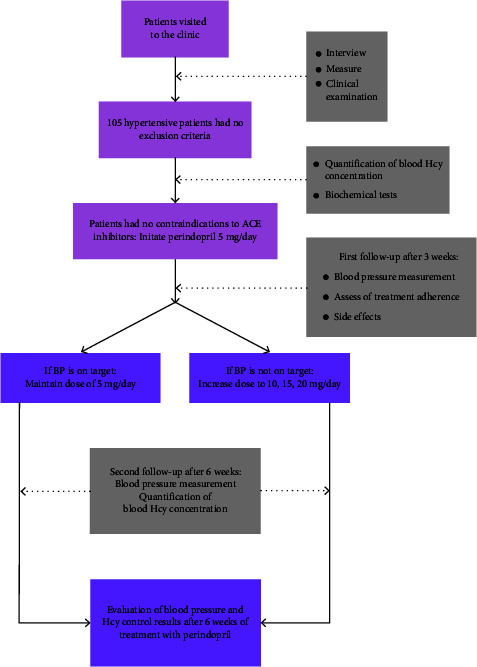
Study diagram.

The sample size was calculated based on the one-proportion sample size estimation formula, *p* = 0.36 is the rate of hyperhomocysteine in hypertensive patients according to the study of Minna Cheng et al. [16]. In fact, we conducted the study on 105 subjects (Figure 1).

#### 2.2.3. Data Collection

All patients who met the JNC 6 criteria for hypertension and had no exclusion criteria were treated for hypertension using JNC 8 strategy A, starting with perindopril 5 mg/day, followed by perindopril 5 mg/day. Then, if blood pressure has reached the target (SBP <140 mmHg and DBP <90 mmHg), we then maintain the dose, and if it does not reach the target, the dose can be increased to 10, 15, and 20 mg/day [16]. The antihypertensive drug of choice was perindopril, trade name Coversyl 5 mg or Coversyl 10 mg from Servier, France. The patient had a first follow-up visit after 3 weeks and a second checkup after 6 weeks to assess blood pressure, treatment adherence, and drug side effects. Evaluation of results after 6 weeks of treatment was as follows: BP control was defined as BP <140/90 mmHg [16], and homocysteine control was achieved when blood homocysteine concentrations were <15 *μ*mol/L [15, 17, 18].

#### 2.2.4. Data Analysis

The mean fasting total Hcy concentration (X ® + SD) has a normal range of 5-<15 *μ*mol/L, defined as increased as ≥15 *μ*mol/L [15]. Age was determined by subtracting the year of birth from the year of study and then divided into 2 groups of ≥60 years and <60 years. Gender is divided into 2 groups as male and female. Diabetes is diagnosed based on the ADA 2017 criteria (similar to the ADA 2021 criteria) if one of the following conditions is met: HbA1C ≥6.5% or fasting blood glucose ≥7.0 mmol/L (126 mg/dL) or random plasma glucose ≥200 mg/dL (11.1 mmol/L) in patients with classic symptoms of hyperglycemia (polyphagia, polydipsia, polyuria, and weight loss) or the patient has been diagnosed with diabetes and is taking medication [19, 20]. Hypertension is divided into 3 grades according to JNC 6 criteria [11], such as the classification of ESC 2021 [12]. Controlled blood pressure is defined as blood pressure below 140/90 mmHg [16]. Controlled blood homocysteine is determined if its concentrations are below 15 *μ*mol/L [15, 17, 18].

#### 2.2.5. Measurements

Blood pressure was measured using a Japanese ALPK2 watch and stethoscope. During the measurement, the patient rested for 15 minutes and refrained from using stimulants and talking. SBP corresponds to the first pulse sound (phase I Korotkoff), and DBP corresponds to its disappearance (phase V Kororkoff). Three measurements of blood pressure were taken 2–5 minutes apart, and the average of the three readings was obtained [21]. Using polarized fluorescence and the principle of competitive immunoassay, the Abbott Diagnostics AxSYM instrument quantified the blood Hcy concentration based on the theory of competitive immunoassay [22]. Glucose concentration was determined using Cobas e automatic biochemical analysis by the enzymatic colorimetric technique [23, 24]. The quantification of HbA1C via the plasma turbidity immunoassay technique was performed by utilizing ARCHITECT i2000R [25].

#### 2.2.6. Statistical Analysis

Computerized data processing was conducted using SPSS 20.0 software. Frequencies and percentages (%) are used to represent qualitative variables (%). To compare the difference between qualitative variables, we utilized the chi-squared test and adjusted according to Fisher's exact test for tables with more than 25 percent expected value <5. In case the measured variables in the paired *t*-test are binary, we use the McNemar test to evaluate the difference between the two groups. To compare the difference between the mean concentration of homocysteine before and after treatment, we used the paired *t*-test. A *p* value of less than 0.05 was considered statistically significant.

## 3. Results

### 3.1. General Subject Characteristics

The study included 105 newly diagnosed primary hypertensive patients with an average age of 63.07 ± 9.32 and a mean Hcy concentration of 17.88 ± 6.03. In which, the prevalence of grade 1 hypertension was 60.9%, grade 2 hypertension was 17.1% and grade 3 hypertension was 21.9%, the rate of raised blood Hcy levels was 74.3%, men represented for 27.6%, age ≥60 represented for 68.6%, and diabetes represented for 46.7% (Table 1).

### 3.2. Results of Blood Pressure Control with Perindopril in Newly Diagnosed Hypertensive Patients

**Figure 2:**
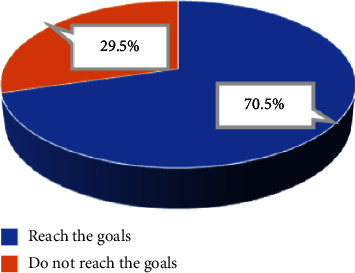
The rate of blood pressure control in the target range after treatment with perindopril.

**Figure 3:**
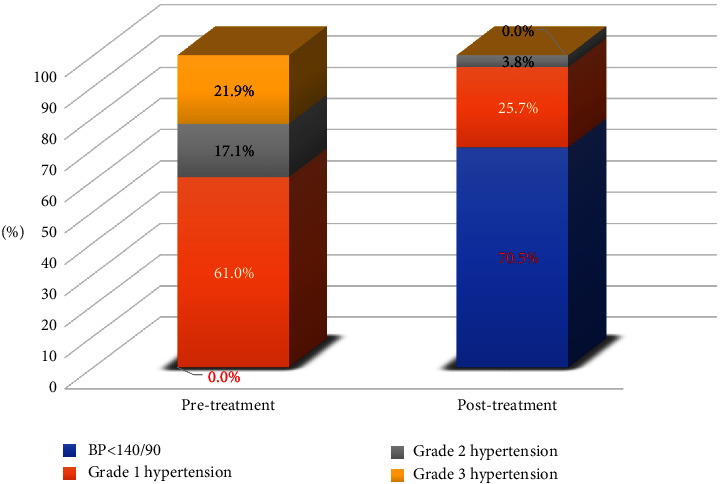
Classification of hypertension before and after treatment with perindopril.

The proportion of patients with BP control below 140/90 mmHg after 6 weeks of treatment with perindopril was 70.5% (Figure 2). After treatment, the proportion of patients with normal blood pressure accounted for 70.5%, grade 1 hypertension decreased from 61.0% to 25.7%, grade 2 hypertension decreased from 17.1% to 3.8%, and no case of grade 3 hypertension was reported (Figure 3). No statistically significant differences were found in the rate of BP control between men and women, between age groups, and between groups with and without diabetes (*p*  >  0.05). However, the rate of BP control in the group of patients with controlled Hcy concentration was higher than in the group of patients without controlled Hcy concentration, and this difference was statistically significant (*p*  <  0.05) (Table 2).

### 3.3. Results of Controlling Blood Homocysteine Levels in Newly Diagnosed Hypertensive Patients after Treatment with Perindopril

**Figure 4:**
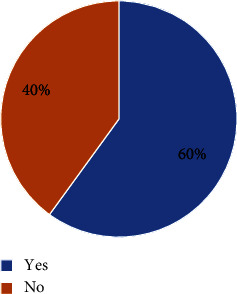
The rate of control blood Hcy concentrations below 15 *μ*mol/L after treatment with perindopril.

**Figure 5:**
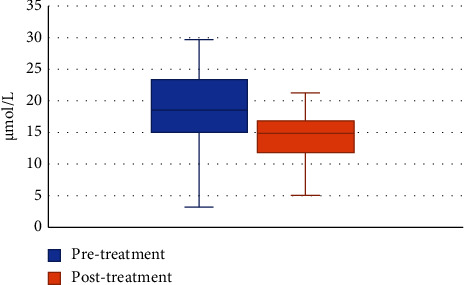
Comparison of blood Hcy levels before and after treatment with perindopril.

The rate of blood Hcy levels below 15 *μ*mol/L after treatment was 60% (Figure 4). The differences were not statistically significant in the rate of Hcy control after treatment between men and women, between age groups, and between diabetic and nondiabetic patients (*p*  >  0.05) (Table 3). At the start of treatment, the rate of control of blood Hcy levels was 25.7%. After 6 weeks of treatment with perindopril, this rate increased from 25.7% to 60%, and this difference was statistically significant (*p* < 0.001). Similarly, when conducting stratified analysis by sex, age groups, and diabetes, we found that the rate of control of blood Hcy levels after treatment rose, and the difference was statistically significant compared to before treatment (*p*  <  0.05) (Table 4). At the start of treatment, the rate of control of blood Hcy levels was 25.7%. After 6 weeks of treatment with perindopril, this rate increased from 25.7% to 60%, and this difference was statistically significant (*p*  <  0.001). Similarly, when conducting stratified analysis by sex, age groups, and diabetes, we found that the rate of control of blood Hcy levels after treatment rose, and the difference was statistically significant compared to before treatment (*p*  <  0.05) (Table 5, Figure 5).

## 4. Discussion

### 4.1. General Characteristics of the Study Population

Our study was conducted on 105 subjects with primary hypertension at the hospital of the Can Tho University of Medicine and Pharmacy. The average age of study participants was 63.07 ± 9.32 years, with the youngest individual being 40 years old and the oldest being 83 years old. The majority of the study participants (68.6%) aged 60. This is the age with a high risk of cardiovascular diseases in general and hypertension in particular [26, 27].

The proportion of women in our study was 2.6 times higher than that of men (72.4% versus 27.6%). According to the 2018 Canadian Hypertension Society analysis, the difference in the sex ratio in hypertension is related to genes and the physiology of sex. Especially after the 5th decade of life, the proportion of women with hypertension tends to increase due to the menopause process [28]. This may partly explain the predominance of women in our study.

In our study, the prevalence of diabetes was 46.7%. Multiple research projects from throughout the world have suggested that hypertension and diabetes are interrelated. Insulin resistance in people with type 2 diabetes causes elevated cholesterol and triglyceride levels, disrupts cell-to-cell communication, including signals that regulate blood pressure, stimulates the sympathetic nervous system, and makes the heartbeat faster and the arteries constrict. In addition, it also creates an imbalance between sodium and potassium (thereby causing an increase in blood volume) and calcium and magnesium (resulting in arterial constriction) and simultaneously induces atherosclerosis of the blood arteries, leading to hypertension [27, 29, 30].

Among 105 hypertensive patients participating in the study, up to 78 people had elevated blood Hcy levels ≥15 *μ*mol/L, accounting for 74.3%. This result shows a relatively close relationship between hypertension and blood Hcy levels. Many studies have shown that the Hcy concentration is an independent risk factor for cardiovascular disease, which can act as a promoter of hypertension through mechanisms such as smooth muscle hypertrophy, decreased function of smooth muscle cells, damaged endothelial cells, and vasomotor dysregulation leading to hardening of the vessel wall [31, 32].

### 4.2. Results of Blood Pressure Control with Perindopril in Newly Diagnosed Hypertensive Patients

Recent years have seen an increase in the number of studies examining the influence of antihypertensive medications on blood Hcy levels used to prevent cardiovascular disease [10, 33, 34]. Potential mechanisms by which Hcy may facilitate the development of hypertension include impaired vascular endothelial and smooth muscle cell function [31]. Thus, the presence of endothelial dysfunction may contribute to altered vasomotor regulation. High Hcy reduces nitric oxide-induced vasodilation, increases oxidative stress, stimulates the proliferation of vascular smooth muscle cells, and alters the elastic properties of vascular walls [31]. In this study, we selected perindopril (Coversyl) monotherapy according to JNC 8 strategy A [16], with the main aim of evaluating the effect of perindopril (Coversyl) on homocysteine in primary hypertensive patients. However, when considering the effectiveness of lowering BP with perindopril monotherapy, the rate of achieving the BP target is quite high, accounting for 70.5%; the rate of grade 1 hypertension decreased from 61% to 25.7%, the rate of grade 2 hypertension decreased from 17.1% to 3.8%, and there were no cases of grade 3 hypertension. Likewise, there were no statistically significant discrepancies in the rate of blood pressure management between men and women, between age groups, and between patients with and without diabetes (*p*  <  0.05). However, we found that the rate of BP control in the group of patients who controlled Hcy <15 *μ*mol/L was higher than in the other group with statistical significance (*p*  <  0.05). Initially, these data indicate that Hcy-controlled hypertensive patients had a better rate of BP-targeted control than hypertensive patients with uncontrolled Hcy. These data enable us to hypothesize that if hypertension individuals are accompanied by a rise in Hcy, it will be more challenging to attain desired blood pressure management. In other words, high blood Hcy levels influence the outcome of each patient's hypertension treatment. Perhaps, additional scientific studies with larger sample sizes and sufficient follow-up time are required to clarify this relationship.

### 4.3. Results of Controlling Blood Homocysteine Levels in Newly Diagnosed Hypertensive Patients after Treatment with Perindopril

Perindopril or ACE inhibitors inhibit ACE activity, resulting in a decrease in angiotensin II. These medicines may also inhibit the breakdown of bradykinin, resulting in a rise in plasma bradykinin, and increase bradykinin-induced vasodilation, resulting in vasodilation and a reduction in vascular pressure [35]. It has been demonstrated that Hcy, via the methionine activation route, increases ACE levels, oxidative stress, and vascular endothelial function, hence increasing blood pressure [36]. In light of these pathophysiological pathways, ACE inhibitors will reduce blood Hcy levels. After six weeks of treatment with perindopril, the proportion of patients with blood Hcy levels ≤15 mol/L increased by 34.3% compared to pretreatment (*p*  <  0.05). The difference in the rate of Hcy control following treatment by gender, age groups, and patients with and without diabetes did not reach statistical significance (*p*  >  0.05). Stratified analyses by gender, age groups, and between groups of diabetic and nondiabetic patients revealed statistically significant differences (*p*  <  0.001) in the rate of control of blood Hcy concentration <15 *μ*mol/L before and after treatment. The mean blood Hcy concentration after treatment was 4.33 *μ*mol/L, which was lower than before treatment, with a 95% confidence interval of 3.69–4.97. The difference was statistically significant at *p*  <  0.001. For each sex, each age group, and between groups of patients with and without diabetes, these differences in concentrations were also significant at *p*  <  0.05.

Poduri et al. also conducted a case-control research study with 273 hypertension patients and 103 control people in order to examine the effect of the medication on plasma Hcy levels. In hypertensive individuals, ACE inhibitors and *β*-blockers dramatically decreased plasma Hcy concentrations, but hydrochlorothiazide considerably increased plasma Hcy concentrations. In this investigation, the Hcy levels before and after 6 weeks of treatment with 5 mg of ramipril were 19.12 ± 6.94 and 14.39 ± 5.75 *μ*mol/L, respectively, with a *p* value less than 0.01. Consequently, their findings imply that ACE inhibitors, particularly ramipril, may be useful in treating hypertension individuals by lowering Hcy levels [10]. After 6 weeks of medication, our study demonstrates that perindopril is likewise successful at controlling Hcy levels.

In addition, several studies examining the effect of ACE inhibitors on various Hcy levels have yielded contradictory results. Šebeková et al. observed the antioxidant effects of ACE inhibitors in patients with nondiabetic kidney disease on short-term use of ramipril, where Hcy was used as a parameter to evaluate oxidative stress. Ramipril (2.5–5.0 mg/day) was administered to 12 newly diagnosed patients for two months, and the data were compared with those of a group of patients (*n* = 7) treated with conventional therapy (diuretics/*β*-blockers). The results showed that the Hcy concentration remained unaffected [37]. Fan et al. studied the change in Hcy levels of 130 subjects with mild and moderate hypertension after 8 weeks of enalapril treatment. Similar to the study above, the authors did not find an increase or decrease in Hcy levels. But stratifying by baseline Hcy levels, the authors found that those with Hcy concentrations <10 *μ*mol/L had a significant increase in plasma Hcy levels (*p* = 0.02) [9]. These inconsistent results could be explained by differences in the individual baseline Hcy levels in each study.

It is probable that the effect of ACE inhibitors on blood Hcy levels has not been demonstrated due to the small sample sizes in the aforementioned research. However, as indicated, in the study by Poduri et al., hypertension individuals treated with ramipril (5 mg/day) for six weeks experienced a significant reduction in Hcy levels. Hcy levels before and after ramipril treatment were 19.12 ± 6.94 *μ*mol/L and 14.39 ± 5.75 *μ*mol/L, respectively [10]. Similarly, Zhao et al. evaluated the impact of enalapril 10 mg in combination with folic acid on blood Hcy levels in 456 patients with moderate and severe hypertension. They found that there was a significant decrease in the plasma Hcy level compared to the baseline with statistical significance *p*  <  0.001 and more pronounced in the enalapril + folic acid combination treatment group than in the enalapril monotherapy group [34]. Fu et al. conducted research on 273 hypertension individuals in China in 2009, dividing them into three subgroups: enalapril 10 mg, enalapril 10 mg + folic acid 0.4 mg, and enalapril 10 mg + folic acid 0.8 mg. After eight weeks of treatment, blood Hcy levels in three groups reduced by 43.8%, 58.5%, and 70.9%, respectively, with statistical significance *p*  <  0.01 [38]. In our study, in both groups Hcy ≥15 *μ*mol/L and Hcy <15 *μ*mol/L after 6 weeks of treatment with perindopril, there was a significant reduction in blood Hcy levels (*p*  <  0.001). Perindopril leads to the reduction of homocysteine, a risk factor for hypertension in particular and cardiovascular disease in general, as demonstrated by our findings. Our findings corroborate the efficacy and role of perindopril in achieving the goal of cardiovascular protection via the Hcy lowering pathway.

### 4.4. Limitations

Despite the fact that our study demonstrated the efficacy of perindopril in lowering blood pressure and homocysteine levels, there are some limitations. This is a cross-sectional study using a single center. It has not yet been demonstrated that lowering homocysteine in the blood improves cardiovascular outcomes and mortality in hypertension patients, as the follow-up duration is still short. We lacked sufficient samples to evaluate the effect of each dose group of perindopril on homocysteine levels in blood. Therefore, additional research with bigger sample sizes, longer follow-up periods, and multicenter, double-blind designs is required to better elucidate the study's limitations and results.

## 5. Conclusion

Perindopril has the ability to lower blood homocysteine levels and regulate blood pressure in patients with primary hypertension.

## Figures and Tables

**Table 1:** General characteristics of the study population.

Characteristics	Mean ± SD or *n* (%)
Age (year)	63.07 ± 9.32
Blood Hcy concentration before treatment (*μ*mol/L)	17.88 ± 6.03
Grade 1 hypertension	64 (60.9)
Grade 2 hypertension	18 (17.1)
Grade 3 hypertension	23 (21.9)
Increased blood Hcy concentration	78 (74.3)
Male	29 (27.6)
Age ≥60	72 (68.6)
Diabetes	49 (46.7)

**Table 2:** The rate of blood pressure controlled by gender, age groups, diabetes, and homocysteine control results.

		Control blood pressure below 140/90 mmHg		OR (95% CI)	*p*
		Yes *n* (%)	No *n* (%)		
Gender	Male	18 (62.1)	11 (37.9)	0.541 (0.221–1.322)	0.243
	Female	56 (73.7)	20 (26.3)		

Age	≥60	48 (66.7)	24 (33.3)	0.75 (0.302–1.862)	0.693
	<60	24 (72.7)	9 (27.3)		

Diabetes	Yes	28 (62.2)	17 (37.8)	0.599 (0.261–1.375)	0.317
	No	44 (73.3)	16 (26.7)		

Control Hcy level below 15 *μ*mol/L	Yes	49 (77.8)	14 (22.2)	2.38 (1.01–5.59)	0.045
	No	25 (59.5)	17 (40.5)		

Chi-square test.

**Table 3:** The rate of control blood Hcy concentrations after treatment by gender, age groups, and diabetes.

		Control blood Hcy level below 15 *μ*mol/L		*p*
		Yes *n* (%)	No *n* (%)	
Gender	Male	13 (44.8)	16 (55.2)	0.082
	Female	50 (65.8)	26 (34.2)	

Age	≥60	40 (55.6)	32 (44.4)	0.247
	<60	23 (69.7)	10 (30.3)	

Diabetes	Yes	31 (63.3)	18 (36.7)	0.523
	No	32 (57.1)	24 (42.9)	

Chi-square test.

**Table 4:** McNemar test comparing the control rate of blood Hcy concentration before and after treatment with perindopril.

Hcy level			Control blood Hcy level below 15 *μ*mol/L after treatment		Total	*p*
			Yes *n* (%)	No *n* (%)		
Yes			26 (24.7)	1 (1)	27 (25.7)	<0.001
No			37 (35.3)	41 (39)	78 (74.3)	
Total			63 (60.0)	42 (40.0)	105 (100)	

Gender	Male	Yes	3 (10.3)	0 (0.0)	3 (10.3)	0.002
		No	10 (34.5)	16 (55.2)	26 (89.7)	
		Total	13 (44.8)	16 (55.2)	29 (100)	
	Female	Yes	23 (30.3)	1 (1.3)	24 (31.6)	<0.001
		No	27 (35.5)	25 (32.9)	52 (68.4)	
		Total	50 (65.8)	26 (34.2)	76 (100)	

Age	≥60	Yes	15 (20.8)	1 (1.4)	16 (22.2)	<0.001
		No	25 (34.7)	31 (43.1)	56 (77.8)	
		Total	40 (55.6)	32 (44.4)	72 (100)	
	<60	Yes	11 (33.3)	0 (0.0)	11 (33.3)	<0.001
		No	12 (36.4)	10 (30.3)	22 (66.7)	
		Total	23 (69.7)	10 (30.3)	33 (100)	

Diabetes	Yes	Yes	15 (33.3)	1 (2.2)	16 (35.6)	0.001
		No	14 (31.1)	15 (33.3)	29 (64.4)	
		Total	29 (64.4)	16 (35.6)	45 (100)	
	No	Yes	11 (18.3)	0 (0.0)	11 (18.3)	<0.001
		No	23 (38.3)	26 (43.3)	49 (81.7)	
		Total	34 (56.7)	26 (43.3)	60 (100)	

^∗^McNemar test.

**Table 5:** Comparison of blood Hcy levels before and after treatment with perindopril.

		Homocysteine	*n*	Mean ± SD	Mean difference 95% CI	*p*
Homocysteine		Pretreatment	105	17.88 ± 6.03	4.33 (3.69–4.97)	<0.001
		Posttreatment	105	13.55 ± 4.01		

Gender	Male	Pretreatment	29	19.98 ± 4.68	4.86 (3.65–6.07)	<0.001
		Posttreatment	29	15.12 ± 3.07		
	Female	Pretreatment	76	17.08 ± 6.31	4.12 (3.36–4.89)	<0.001
		Posttreatment	76	12.96 ± 4.18		

Age	≥60	Pretreatment	72	18.61 ± 5.72	4.52 (3.76–5.28)	<0.001
		Posttreatment	72	14.09 ± 3.92		
	<60	Pretreatment	33	16.28 ± 6.44	3.91 (2.68–5.15)	<0.001
		Posttreatment	33	12.37 ± 4.02		

Diabetes	Yes	Pretreatment	49	17.31 ± 6.59	4.07 (3.10–5.03)	<0.001
		Posttreatment	49	13.24 ± 4.31		
	No	Pretreatment	56	18.38 ± 5.49	4.56 (3.68–5.43)	<0.001
		Posttreatment	56	13.82 ± 3.75		

^*∗*^Paired-sample *t*-test.

## Data Availability

The datasets generated and/or analyzed during the current study are available from the corresponding author on reasonable request.

## Abbreviations

ADA: American Diabetes Association
AHA: American Heart Association
BMI: Body mass index
CRP: C-reactive protein
ESC: European Society of Cardiology
Hcy: Homocysteine
JNC: Joint National Committee
SD: Standard deviation.
